# Time dynamics of symptom progression in patients with acute pancreatitis: a Dynamic Time Warping analysis

**DOI:** 10.3389/fmed.2025.1703268

**Published:** 2025-11-17

**Authors:** Rui Han, Xiaoyu Deng, Hebing Liu, Yujie Duan, Kexin Huang, Qinghuan Kong, Yuhang Pu, Haiqi Yang, Yongliang Jiao, Zhaohua Cheng, Yong Jia

**Affiliations:** 1 School of Nursing, Jilin University, Changchun, China; 2 Department of Physiology, Development and Neuroscience, University of Cambridge, Cambridge, United Kingdom; 3 School of Nursing and Well-Being, Changchun Humanities and Sciences College, Changchun, China; 4 Department of Hepatobiliary and Pancreatic Surgery, The Second Hospital of Jilin University, Changchun, China; 5 School of Sport Health and Technology, Jilin Sport University, Changchun, China

**Keywords:** acute pancreatitis, symptom, dynamics, Dynamic Time Warping (DTW), network analysis

## Abstract

**Background:**

Acute pancreatitis (AP) morbidity has been increasing in recent years. Patients with AP exhibit highly variable symptom patterns over time, posting challenges to traditional analytical methods. Dynamic Time Warping (DTW) effectively aligns temporal sequences of different rhythms, offering a novel approach to model these complex dynamics.

**Objective:**

This study employs DTW technology to systematically analyze the individualized developmental trajectories of symptom clusters in patients with AP, delving into the heterogeneous characteristics during the process of time series changes.

**Methods:**

In a longitudinal study of 155 patients with AP, 32 symptoms were assessed using the Memorial Symptom Assessment Scale at hospitalization and 1, 3, 6, 9, and 12 months post-discharge. DTW was used to analyze temporal dynamics, generating individual symptom distance matrices. At the group level, these matrices are integrated using Distatis analysis, followed by hierarchical clustering to identify symptom clusters and network analysis to determine central symptoms.

**Results:**

Each patient had unique symptom manifestations and dynamic change patterns. Six major symptom clusters were identified: emotional disorder cluster, appetite disorder cluster, multi-system physical discomfort cluster, localized physiological perception abnormality cluster, functional decline cluster, and abdominal discomfort cluster. Centrality analysis revealed that the appetite domain exhibited high centrality, suggesting that its variation may influence multiple aspects of patient experience.

**Conclusion:**

Dynamic Time Warping provides a novel and effective approach for analyzing the temporal trajectories of symptoms both within and across individuals. The research results provide methodological support and empirical evidence for individualized symptom management, early intervention, and predictive model construction of AP progression.

## 1 Introduction

Acute pancreatitis (AP) ranks among the most common prevalent digestive system disorders worldwide, with potential onset across all age groups but predominantly affecting adults. The incidence of AP ranges from 30 to 100 per 100,000 population and continues to rise annually (1, 2). Approximately 20% of patients develop moderate to severe AP (grade II-III), associated with a mortality rate of 20%–40% (3). In the United States, each AP-related hospitalization incurs an average cost of $9,870, amounting to $2.2 billion annually (4). While overall medical costs for AP in China have declined, expenses related to severe cases remain essentially unchanged (5). AP imposes substantial physical and psychological burdens, prolongs hospital stays, increases financial strain, and adversely affects patients’ quality of life and family well-being.

The concept of symptom clusters has evolved significantly since its initial formalization. In Dodd et al. (6) first defined a symptom cluster as a constellation of at least three concurrent and interacting symptoms. In Kim et al. (7) broadened this definition to include two or more co-occurring, interrelated symptoms that are distinct from other clusters. Aktas et al. (8) proposed that symptom clusters comprise at least two symptoms with strong internal associations and greater intra-cluster than inter-cluster correlations. Although no universally accepted definition exists, symptom clusters are generally understood as groups of interrelated, simultaneously occurring symptoms. Research in this field is crucial, as clusters often exert a greater impact on patients than individual symptoms. Unlike single-symptom approaches, symptom cluster management better reflects the complexity of real-world clinical practice. Identifying and assessing clusters enhances understanding of symptom interrelationships, enabling clinicians to detect overlooked symptoms, perform more comprehensive evaluations, and address multifaceted clinical challenges more effectively within limited time frames.

Acute pancreatitis typically features sudden-onset upper abdominal pain, frequently accompanied by nausea, vomiting, and abdominal distension. In severe cases, symptoms may progress to dysphoria, jaundice, indigestion, hypotension, or even shock. Machicado et al. (9) demonstrated that compared with individuals of the same gender and similar age without pancreatitis, AP survivors exhibited reduced long-term health-related quality of life (HRQoL). Bejjani et al. (10) reported that 57% of participants experienced persistent gastrointestinal symptoms 12 months after acute pancreatitis, including abdominal pain, frequent diarrhea, and distress from consuming greasy foods. A Japanese study (11) showed that the most common initial symptoms in patients were abdominal pain (92.1%), followed by vomiting (27.0%), fever (16.9%), and back pain (16.7%). In their qualitative analysis, Ma et al. (12) conceptualized patients’ psychological responses through three interconnected themes: perceived disease unpredictability, stress-coping dynamics, and adaptive capacity development. A population-based study (13) established anxiety prevalence of 29% and depression of 35.7% in acute pancreatitis cohorts, confirming that biopsychosocial symptom burdens significantly exacerbate disease progression and worsen clinical outcomes. AP represents a clinically heterogeneous disorder (2) with dynamic progression and variable trajectories. Without timely intervention, it can easily lead to critical metabolic derangements and multi-organ dysfunction, including gastrointestinal failure, posing significant mortality risks (14, 15). Given its substantial interpatient variability in symptom severity, AP necessitates personalized management strategies (16). To move beyond conventional treatment paradigms, a precision-based diagnostic and therapeutic approach is required – stratified by etiology, disease severity, patient characteristics, and subgroup-specific needs (17). Current research on AP symptoms has primarily focused on the mechanisms, assessment, and management of individual symptoms, while studies addressing symptom clusters remain limited. Despite the growing academic attention to the symptom burden of AP, there is still a notable lack of longitudinal studies capable of capturing the dynamic evolution of symptoms over time. Most existing investigations rely on cross-sectional designs or static assessment methods, which fail to account for the temporal misalignment and individualized progression patterns of symptoms. This limitation impedes our understanding of how symptom clusters interact and evolve throughout the disease trajectory, thereby constraining the development of timely and personalized intervention strategies. Accurate assessment of symptoms is a fundamental prerequisite for effective clinical management. Therefore, longitudinal studies investigating the dynamic trajectories of symptom clusters in AP are imperative for informing patient-centered care and improving quality of life outcomes.

Current symptom research predominantly employs cross-sectional designs (18), implementing correlational techniques such as factor analysis (19), latent class analysis (LCA) (20), hierarchical cluster analysis (21), and structural equation modeling (SEM) (22). These methodologies, however, struggle to model the evolution of symptom trajectory and infer causative mechanisms. While the vector autoregressive (VAR) models offer enhanced capacity for longitudinal dynamic analysis (23), their clinical translation remains limited due to exacting data specifications (24). Principal Component Analysis (PCA) is commonly applied in time-series data analysis (25); however, its reliance on linearity and temporal alignment limits its effectiveness in capturing the complex and asynchronous symptom trajectories observed in AP. Pronounced inter-individual divergences in symptom chronometry fundamentally limit extant methodologies in capturing temporal symptom dynamism and patient-specific trajectory patterns. Moreover, regression-based approaches [e.g., logistic regression (26)] typically reduce time to a static covariate, thereby neglecting the continuous and evolving nature of symptoms. In time-series clustering, discrepancies in sequence length and rhythm can introduce alignment biases, ultimately impairing the accurate identification of symptom patterns.

To address the limitations of traditional methods, Dynamic Time Warping (DTW) emerges as a promising nonlinear alignment technique (27, 28). First proposed by Berndt and Clifford (29), DTW quantifies shape-based similarity in time series data by allowing flexible temporal alignment, originally developed for speech recognition (30), and later gradually expanded to signature verification (31), medical diagnosis (32), and psychiatric research (33–37). DTW can address various issues in time series data. This enables the comparison of symptom trajectories among individuals within the same temporal framework, aiding in the identification of patterns of symptom change and addressing the issues of different starting points and progression differences. Consequently, it facilitates the identification of conserved symptom transition signals and effectively addresses confounders arising from varying symptom onset timing and progression dynamics. For nonlinear symptom trajectories, DTW can match and align symptom changes with different rhythms and patterns, aiding in the analysis of symptom volatility and complexity. For multi-symptom comorbidity analysis, DTW can simultaneously handle the variation trajectories of multiple symptoms, aiding clinicians in identifying the interactions between different symptoms and revealing their mutual influences and intrinsic connections. Furthermore, DTW can compare the symptom trajectories of different patients, identify similarities and differences, and reveal the dynamic connections and development patterns between symptoms. This approach can identify potential high-risk groups or assess the effects of interventions, and also help identify the interactions between symptoms and core symptoms. In clinical settings, where symptom assessments are often conducted at irregular intervals and trajectories differ markedly across patients, DTW offers a distinct methodological advantage. It flexibly accommodates inconsistencies in data collection timing that often challenge vector autoregressive (VAR) models. Through its nonlinear alignment capability, DTW can also capture variations in symptom onset and progression. This capability addresses the key limitation of principal component analysis (PCA), which is constrained by linear assumptions and frequently fails to detect such complex temporal patterns.

In this study, DTW was employed to explore longitudinal symptom dynamics in 155 AP patients. Using the Memorial Symptom Assessment Scale (MSAS), 32 symptoms were evaluated at baseline, 1, 3, 6, 9, and 12 months, and proposed individual-level (i.e., idiographic) and group-level (i.e., nomothetic) analyses.

## 2 Materials and methods

### 2.1 Study sample

This 1-year longitudinal observational study enrolled 226 patients diagnosed with AP at a tertiary hospital in Changchun, China. Eligible participants were aged ≥18 years, met the diagnostic criteria for AP as outlined in the Chinese Guidelines for Diagnosis and Treatment of Acute Pancreatitis (2021) (38), and provided written informed consent. Exclusion criteria comprised: (1) comorbid severe organ dysfunction (cardiac, hepatic, pulmonary, renal failure) or malignant tumors; (2) pregnancy or lactation; (3) history of psychiatric illness or cognitive impairment; (4) communication disorders impeding study participation; (5) prior surgical intervention for AP. Ethical approval was granted by the Ethics Committee of the Second Hospital of Jilin University (Approval No. 031-2023).

Baseline data collection included two main domains. Sociodemographic variables encompassed age, sex, educational level, marital status, place of residence, and monthly income. Disease-related variables comprised etiology, alcohol consumption history, smoking status, comorbidities (including diabetes, gallstones, hyperlipidemia), hospitalization costs, and body mass index (BMI).

Telephone interviews were conducted at 1, 3, 6, 9, and 12 months to assess symptom profiles. Of the 226 patients initially enrolled for symptom assessment, 155 completed all six waves of follow-up and thus constituted the full analysis cohort. This sample size is comparable to or larger than those reported in prior longitudinal studies using DTW in clinical symptom science (36).

### 2.2 Measurements

The MSAS, developed by Portenoy et al. (39), was employed to evaluate the symptomatic experiences of patients with AP. This instrument assesses physical, psychological, and psychosocial symptoms through validated frequency, severity, and distress metrics. The present study employed the Chinese version of the MSAS (MSAS-Ch), adapted and validated by Cheng et al. (40), to comprehensively evaluate symptoms experienced by patients over the preceding 7 days. The assessment comprised four domains: symptom incidence (presence/absence), frequency rated on a 4-point Likert scale (“rarely” to “almost constantly”), severity measured via a 4-point Likert scale (“mild” to “very severe”), and distress level quantified using a 5-point Likert scale (“not at all” to “very much”). The score for each individual symptom was calculated as the mean of its frequency, severity, and distress.


$$S\,\mathrm{ymptom}\,\mathrm{score}=\frac{\mathrm{frequency}\,+\,\mathrm{severity}\,+\,\mathrm{distress}}{3}$$

### 2.3 Statistical analysis

Baseline sociodemographic and disease-related variables were summarized using descriptive statistics. Categorical variables are presented as frequencies (%). Longitudinal panel data were constructed from 32 symptom scores (MSAS-Ch) collected at uniform time points (baseline, 1, 3, 6, 9, 12 months). Participants completing ≤5 symptom assessments were excluded, yielding a final cohort of 155 individuals for trajectory analysis. The flowchart of inclusion and exclusion criteria is in Supplementary Appendix 1.

**FIGURE 1:**
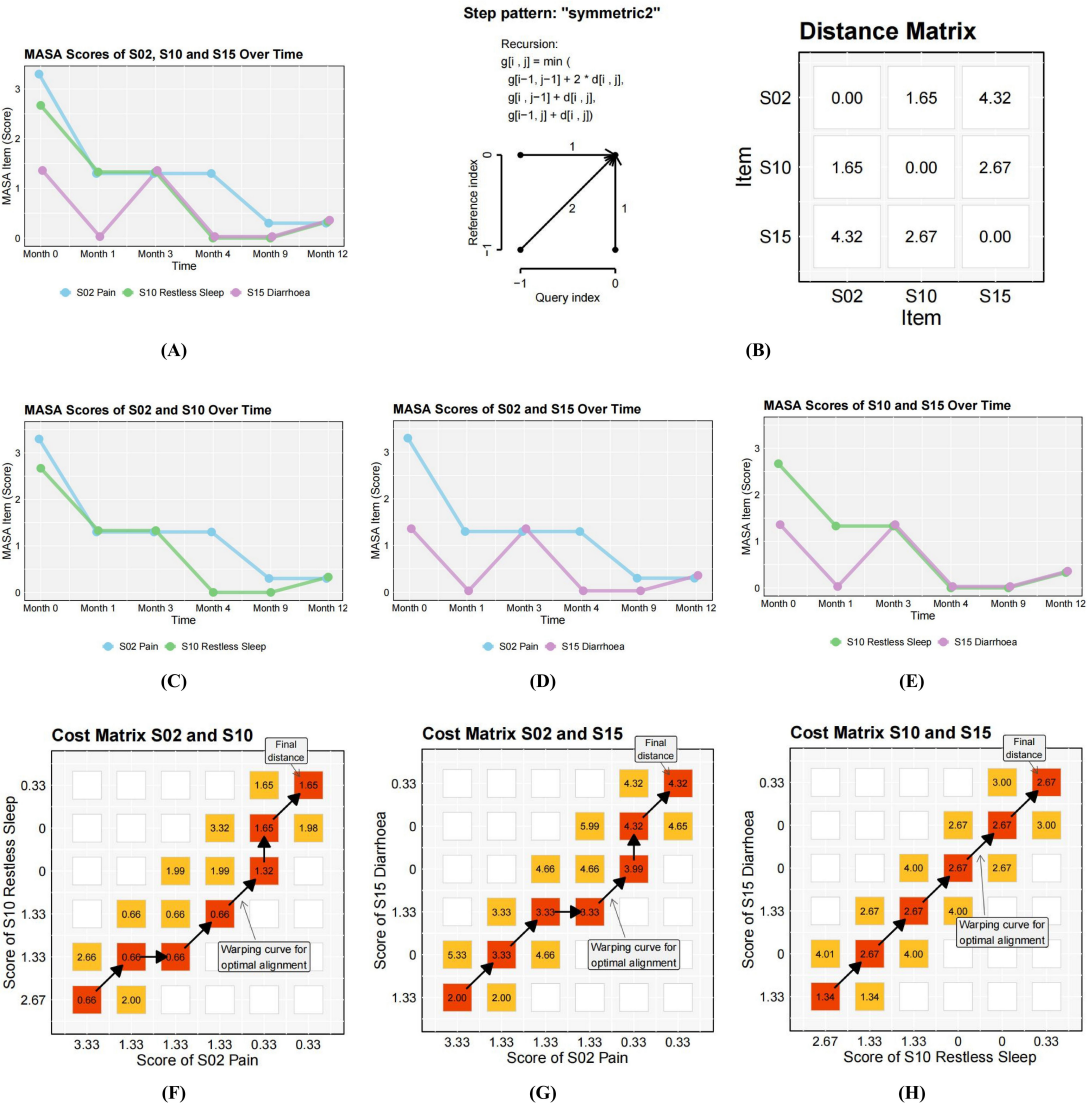
Explanation of the dynamic time warp (DTW) analysis. DTW quantifies similarity between temporal sequences (here, symptom trajectories of three MSAS items). The algorithm first constructs a 6 × 6 local cost matrix (LCM). It then identifies the optimal warping path by traversing the LCM from [1,1] to [6,6] whilst minimizing cumulative alignment cost. **(A)** Depicts MASA scores of S02, S10, and S15 over time. A Sakoe–Chiba bandwidth of 1 and the symmetric2 step pattern **(B)** were applied, following established practices in clinical symptom research (36, 37). This conservative parameter combination prevents physiologically implausible alignments while retaining sensitivity to short-term variations and ensuring unbiased alignment across time series. **(C–E)** demonstrate DTW distance computations for three symptom pairs, yielding distances of 1.65 (S2–S10), 4.32 (S2–S15), and 2.67 (S10–S15). The significantly lower distance for S2–S10 (1.65) indicates greatest temporal similarity. **(F–H)** Illustrate cost matrices and optimal warping paths for S02–S10, S02–S15, and S10–S15.

Dynamic Time Warping (DTW) was employed to compute pairwise symptom distances for each patient, with the algorithmic implementation illustrated in Figure 1. Within this computational framework, smaller inter-symptom distances indicate higher temporal congruence, reflecting greater similarity in symptom trajectories over time. DTW is particularly well-suited for modeling temporally desynchronized symptom patterns due to its capacity for nonlinear alignment via local temporal distortion. The algorithm identifies the optimal warping path through dynamic programming under Bellman’s optimization principle, minimizing the cumulative distance between sequences (41). To control excessive temporal flexibility and reduce computational complexity, a Sakoe-Chiba band with a time-window width of one was applied. This constraint restricts the warping path to a narrow temporal range (t−1, t, t+1), thereby enhancing sensitivity to short-term fluctuations while preserving local temporal structure. To address potential mismatches at the start and end of the time series, linear interpolation was used by inserting five equidistant values between each time point prior to distance computation. The “symmetric2” step pattern was applied to enforce symmetric constraints on point mapping between time series, ensuring structurally consistent alignment.

This approach enabled the generation of a 32 × 32 DTW distance matrix for each patient, capturing all pairwise temporal relationships among symptom items. From each distance matrix, a hierarchical clustering dendrogram and an undirected symptom co-evolution network were subsequently derived, enabling the visualization of temporal proximity and structural clustering patterns. Figure 1 illustrates the analysis framework using three symptom trajectories from a single participant as an example. Symptom trajectories exhibiting similar temporal evolution are characterized by smaller DTW distances. In this illustrated case, the trajectories of symptoms S2 and S10 demonstrate a higher degree of temporal congruence (S02–S10: 1.65) compared to their respective distances from S15 (S2–S15: 2.65; S10–S15: 4.32). This undirected analysis captures symptom co-evolution within individual MSAS profiles.

Dynamic Time Warping analysis performed for each patient yielded 155 individual 32 × 32 matrices. The Distatis method was subsequently applied to derive a group compromise matrix (42), which integrates and analyses multiple distance matrices to elucidate the latent structure characterizing group-level symptom dynamics. Distatis extends PCA to integrate multiple distance matrices by identifying principal components, known as compromise factors, that best capture variance across subjects. The first three compromise factors explained the largest proportion of variance and were used as Cartesian coordinates for plotting the 32 symptoms, where spatial distances between points reflected average temporal dissimilarity, with more closely positioned symptoms exhibiting more similar temporal dynamics. Two-dimensional symptom configurations were visualized through scatter plots of (a) Factor 1 versus Factor 2, (b) Factor 2 versus Factor 3 and (c) Factor 1 versus Factor 3.

To extract a hierarchical clustering structure from the compromise configuration derived via Distatis, the Ward. *D*^2^ method was used to construct dendrograms reflecting symptom similarity. The optimal number of clusters was determined using the elbow method, which evaluates the within-cluster sum of squared errors (SSE) across varying numbers of clusters. The resulting elbow plot identified an inflection point where the rate of SSE reduction substantially decreased, corresponding to the point of maximal curvature change between adjacent segments. This point was interpreted as the optimal trade-off between model complexity and explanatory power. In the present analysis, this approach identified six principal symptom clusters.

For the directed analysis, the DTW algorithm was implemented with an asymmetric Sakoe-Chiba band constraint (43), enforcing unidirectional alignment (e.g., from Symptom A to Symptom B only). This procedure yielded a directed distance matrix for each of the 155 individuals, capturing the directional temporal relationships among symptom dimensions. A group-level directed network was then constructed by averaging these individual matrices, producing a summary representation of directional symptom dynamics across the cohort. From the aggregated network, in-strength and out-strength centrality metrics were computed to quantify the directional influence of each symptom. Symptoms exhibiting significant out-strength centrality denote those whose fluctuations typically precede changes in other symptoms, whereas symptoms with elevated in-strength centrality consistently follow corresponding fluctuations in other symptoms (36).

Descriptive analyses were performed using IBM SPSS Statistics version 25. Dynamic time warping and network analyses were conducted in R (version 4.4.3; R Foundation for Statistical Computing, Vienna, Austria, 2016)^1^ through RStudio. The main R packages employed were “dtw” (version 1.22-3) for trajectory alignment and “qgraph” (version 1.6.9) for network visualization and analysis.

## 3 Results

Table 1 details sociodemographic and disease-related variables of the cohort (*N* = 155) at baseline. The cohort was composed predominantly of married (85.2%), urban (63.2%), males (61.9%), with a mean age of 48.2 years (SD = 15.4). In terms of education, the largest group had completed middle school (41.9%). Most reported monthly incomes below 3,000 Yuan (62.6%). Clinical profiles revealed biliary (51.0%) and hyperlipidaemic (32.9%) etiologies. Smoking and alcohol histories were present in 20.0% and 24.5%, respectively. Comorbid gallstones and hyperlipidemia affected 30.3% and 36.1%. The mean body mass index (BMI) was 24.6 kg/m^2^ (SD = 3.9), and 86.5% incurred hospitalization costs under 50,000 Yuan.

**TABLE 1:** Baseline sociodemographic and disease-related variables of 155 patients with acute pancreatitis.

Variables	*N* = 155
Sex, male	96 (61.9%)
Age (years), mean ± SD	48.2 ± 15.4
Education level	
Primary school	30 (19.4%)
Middle school	65 (41.9%)
High school	16 (10.3%)
University and above	44 (28.4%)
Marital status	
Unmarried	23 (14.8%)
Married	132 (85.2%)
Place of residence	
Rural	57 (36.8%)
Urban	98 (63.2%)
Monthly income, Yuan	
<1,000	55 (35.5%)
1,000 to 2,999	42 (27.1%)
3,000 to 4,999	17 (11%)
≥5,000	41 (26.5%)
Etiology	
Biliary	79 (51%)
Hyperlipidemic	51 (32.9%)
Alcoholic	15 (9.7%)
Others	10 (6.5%)
Smoking history	
No	124 (80%)
Yes	31 (20%)
Alcohol consumption history	
No	117 (75.5%)
Yes	38 (24.5%)
Presence of diabetes	
No	121 (78.1%)
Yes	34 (21.9%)
Presence of gallstones	
No	108 (69.7%)
Yes	47 (30.3%)
Presence of hyperlipidemia	
No	99 (63.9%)
Yes	56 (36.1%)
Hospitalization cost, Yuan	
<50,000	134 (86.5%)
50,000 to 100,000	18 (11.6%)
>100,000	3 (1.9%)
Body mass index (BMI, kg/m^2^), mean ± SD	24.6 ± 3.9

### 3.1 Individual analyses (idiographic approach)

**FIGURE 2:**
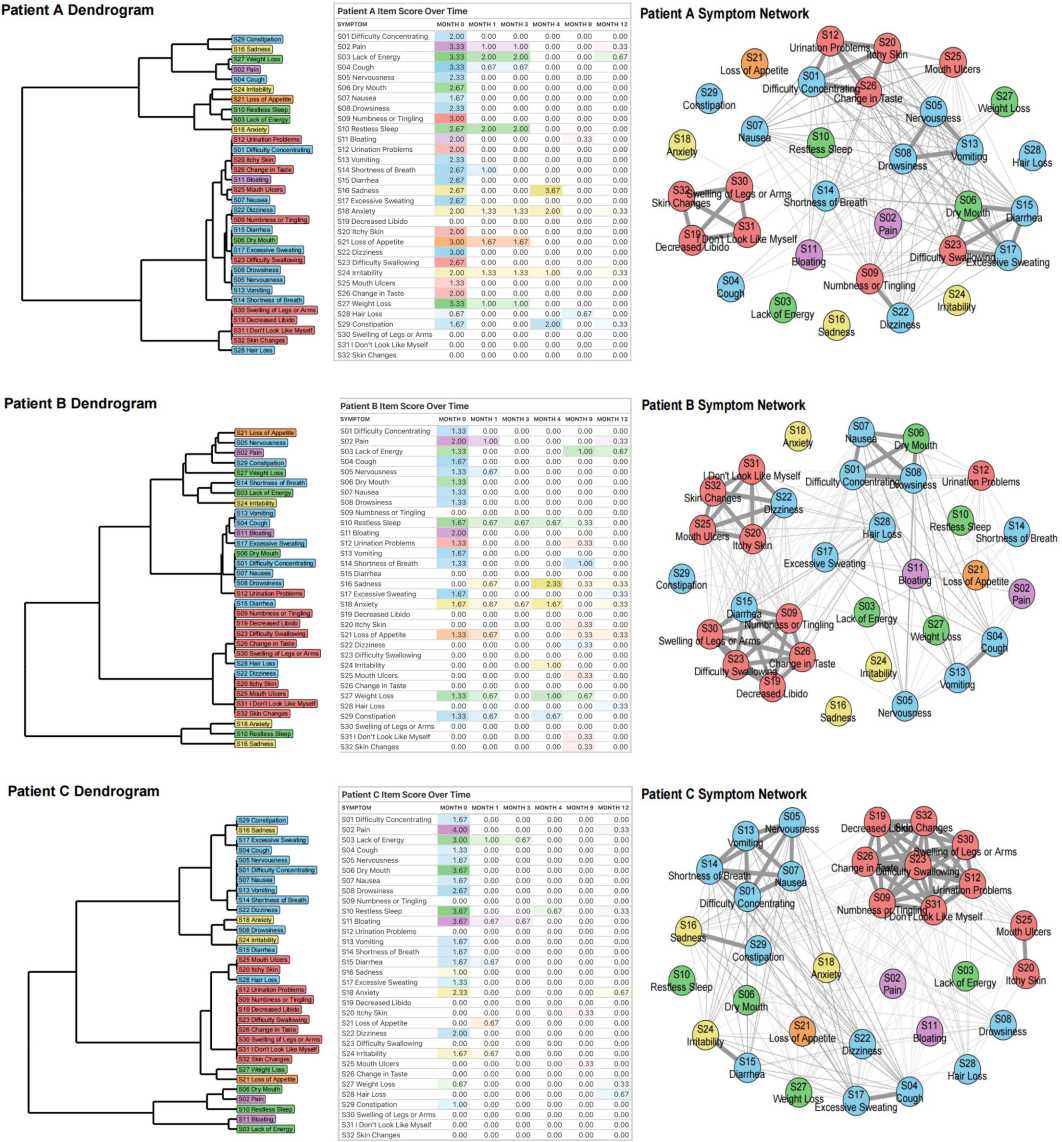
Dynamic Time Warping analysis of three patients (A, B, C). For each participant, we first generated a dendrogram of symptom clusters, reflecting increasingly similar symptom trajectories over time. We then compiled a rating table of raw symptom scores (severity color-coded) across six time periods. Finally, we constructed individual symptom networks using DTW analysis.

The DTW clustering methodology was implemented at the participant level. For each individual, dual 32 × 32 distance matrices were computed: a symmetric matrix (undirected relationships) and an asymmetric matrix (directed relationships), capturing symptom-pair dynamics across six longitudinal assessments. Each matrix element quantifies the pairwise symptom dissimilarity (DTW distance). Per participant, we constructed: (1) a directed symptom network, (2) an undirected symptom network, and (3) a dendrogram representing symptom cluster hierarchies, as shown in Figure 2. To demonstrate individual-level DTW utility, we present three exemplary acute pancreatitis cases, revealing marked heterogeneity in symptom trajectories and clustering patterns. Therefore, we selected 3 participants, whose baseline information is in Supplementary Appendix 2.

In Patient A, certain symptoms demonstrate strong interrelations, such as S01 (difficulty concentrating), S12 (urination problems), S20 (itchy skin), and S26 (change in taste). S26 (change in taste) may affect appetite, leading to insufficient food intake and inadequate energy supply, which can impact brain function and trigger S01 (difficulty concentrating). Meanwhile, S12 (urination problems) may disrupt sleep quality due to frequent nocturia, causing daytime fatigue and exacerbating S01 (difficulty concentrating). If urination problems lead to the accumulation of toxins or metabolic waste products in the body, this may irritate the skin and worsen S20 (itchy skin). In addition, S20 (itchy skin) can distract patients, further exacerbating S01 (difficulty concentrating). These symptoms are interconnected and mutually influential, reflecting the systemic cascade triggered by acute pancreatitis. Another group of interrelated symptoms - S19 (decreased libido), S30 (swelling of legs or arms), S31 (“I don’t look like myself”), and S32 (skin changes) - all belong to the localized physiological perception abnormality cluster and reflect a self-reinforcing cycle driven by endocrine dysfunction and impaired nutrient absorption. Impaired pancreatic endocrine function disrupts sex hormones, decreasing libido and affecting psychological well-being. Concurrently, malnutrition-induced hypoproteinemia lowers plasma colloid osmotic pressure, causing fluid extravasation into interstitial spaces. This manifests as S30 (swelling of legs or arms), leading to discomfort, mobility issues, and infection risk. Furthermore, both S30 (swelling of legs or arms) and S32 (skin changes) can trigger S31 (“I don’t look like myself”), causing anxiety and depression that compromise mental health. A third group - S05 (nervousness), S08 (drowsiness), S13 (vomiting) - comprises symptoms within the multi-system physical discomfort cluster. Vomiting, driven by pancreatic inflammation, can cause dehydration and electrolyte disturbance, exacerbating physical discomfort and tension. Nervousness may amplify nausea and disrupt sleep, contributing to drowsiness. These interactions illustrate the internal coherence and systemic burden of this cluster.

Compared to patient A, patient B has other symptom connections, such as S01 (difficulty concentrating), S06 (dry mouth), S07 (nausea) and S08 (drowsiness) being closely linked. Digestive enzymes secreted by the pancreas are activated, stimulating the gastrointestinal tract and causing nausea. Nausea may promote vomiting, further aggravating physical discomfort and drowsiness. Dry mouth may be caused by dehydration or electrolyte imbalance. Frequent nausea and vomiting can lead to fluid loss, causing dehydration and dry mouth. Dry mouth not only increases the patient’s discomfort, but may also affect the patient’s appetite and swallowing ability, further aggravating nutritional absorption disorders. Drowsiness and dry mouth may also further aggravate difficulty concentrating due to physical discomfort and dehydration.

Like patients A and B, symptoms within patient C’s localized physiological perception abnormality cluster are closely interconnected. In addition, Patient C’s S01 (difficulty concentrating), S05 (nervousness), S07 (nausea), S13 (vomiting), and S14 (shortness of breath) are closely related, and they all belong to the multi-system physical discomfort cluster. They collectively reflect the multi-system effects of AP. Systemic inflammatory response leads to discomfort such as nausea, vomiting, and shortness of breath. These symptoms interact to form a vicious cycle, further triggering tension and poor concentration, while shortness of breath may also exacerbate attention problems due to hypoxia.

The close correlation of these symptoms suggests that physicians need to comprehensively assess the patient’s condition and adopt comprehensive treatment measures to alleviate symptoms and prevent further deterioration of the condition.

### 3.2 Group-level analysis (nomothetic approach)

**FIGURE 3:**
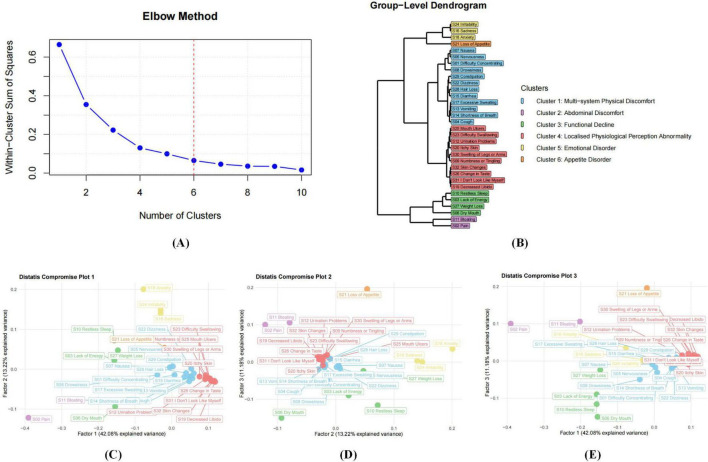
Nomothetic analyses based on all distance matrices from 155 participants. **(A)** shows that the number of dimensions (symptom clusters) in the data is determined using an elbow plot, which is based on the eigenvalues in the downward curve of the three compromise factors. **(B)** displays the dendrogram of the hierarchical clustering process based on the three compromise factors. **(C–E)** display the compromise plots based on Distatis analysis. These represent the positions of the 32 MSAS items in the compromise space using the first 2 compromise factors **(C)**, the second and third compromise factors **(D)** and the first and third compromise factors **(E)**. The white horizontal and vertical error lines indicate the 95% confidence intervals, estimated using bootstrap with 500 resampling samples.

Figure 3 presents a dimensional analysis of symptom data from 155 patients with AP at baseline. According to the elbow plot (Figure 3A), six dimensions were identified as the optimal solution. DTW and subsequent Distatis analysis yielded three compromise factors, explaining 28.5%, 14.1%, and 10.2% of the total variance, respectively. Using hierarchical clustering, informed by the dendrogram (Figure 3A), six major symptom clusters were derived: the emotional disorder cluster (3 items): S24 (irritability), S16 (sadness), S18 (anxiety); the appetite disorder cluster (1 item): S21 (loss of appetite); the multi-system physical discomfort cluster (12 items): S07 (nausea), S05 (nervousness), S01 (difficulty concentrating), S08 (drowsiness), S29 (constipation), S22 (dizziness), S28 (hair loss), S15 (diarrhea), S17 (excessive sweating), S13 (vomiting), S14 (shortness of breath), S04 (cough); the localized physiological perception abnormality cluster (10 items): S25 (mouth ulcers), S23 (difficulty swallowing), S12 (urination problems), S20 (itchy skin), S30 (swelling of legs or arms), S09 (numbness or tingling), S32 (skin changes), S26 (change in taste), S31 (“I don’t look like myself”), S19 (decreased libido); the functional decline cluster (4 items): S10 (restless sleep), S03 (lack of energy), S27 (weight loss), S06 (dry mouth); the abdominal discomfort cluster (2 items): S11 (bloating), S02 (pain).

**FIGURE 4:**
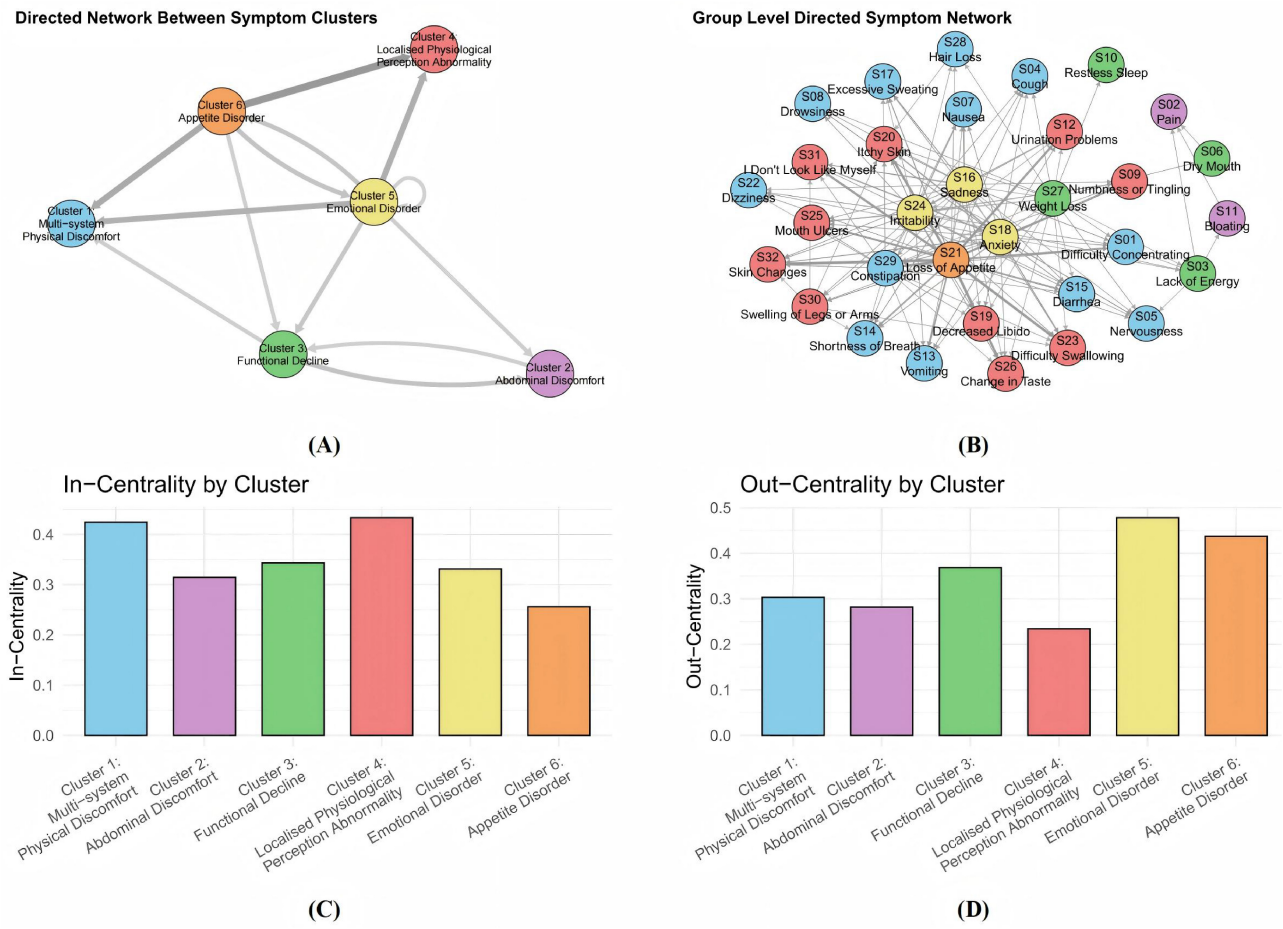
Directed symptom network of 155 AP patients. **(A)** A directed network among symptom clusters. Nodes of different colors represent distinct symptom clusters, the direction of the arrows indicates the influential relationships between these clusters. **(B)** A group - level directed symptom network. The dense nodes and complex connections demonstrate the directed associations between various specific symptoms. **(C)** The dimensions localised physiological perception abnormality cluster and multi-system physical discomfort cluster have the strongest in-strength centrality. **(D)** The emotional disorder cluster has the strongest out-strength centrality. The centrality metrics quantify a symptom’s role: out-centrality identifies symptoms that act as potential drivers, whose changes precede and influence those of other symptoms, while in-centrality identifies symptoms that function as potential consequences, whose changes tend to follow alterations in other symptoms.

The directed network diagram in Figure 4 revealed that both the emotional disorder cluster and the appetite disorder cluster exhibit high out-centrality, indicating their strong driving influence on other symptom clusters in AP. Emotional symptoms such as sadness and anxiety, often arising from physical discomfort and psychological stress, can aggravate other symptoms by amplifying pain, impairing sleep, and weakening immune response. Similarly, loss of appetite contributes not only to malnutrition and energy deficits but also to the aggravation of a wide range of symptoms through systemic physiological consequences. Together, these two clusters act as upstream nodes within the symptom network, playing pivotal roles in driving disease progression and shaping the overall symptom trajectory.

In contrast, the multi-system physical discomfort cluster and the localized physiological perception abnormality cluster exhibited significant in-centrality, indicating that they are key convergence nodes in the network. This suggests that they are particularly sensitive to upstream influences, with symptom aggravation in other clusters likely to propagate into and intensify manifestations within these two clusters. Their responsiveness to network-wide symptom dynamics indicates their potential utility as early warning indicators of clinical deterioration and highlights the need for close monitoring. The multi-system physical discomfort cluster, in particular, appears to reflect the overall disease burden. It may contribute to the worsening of symptoms in the functional decline cluster through systemic inflammation, which disrupts both endocrine and exocrine pancreatic function. Progressive pancreatic impairment may further reinforce this systemic burden: reduced insulin secretion leads to hyperglycemia, which impairs multiple organ systems and exacerbates physical discomfort, while exocrine insufficiency results in maldigestion, further affecting abdominal discomfort. Patients commonly report bloating and diarrhea. These interconnections are reflected in the high in-strength centrality of the multi-system physical discomfort cluster, underscoring its vulnerability to symptom propagation and its importance in the evolving clinical profile of acute pancreatitis.

**FIGURE 5:**
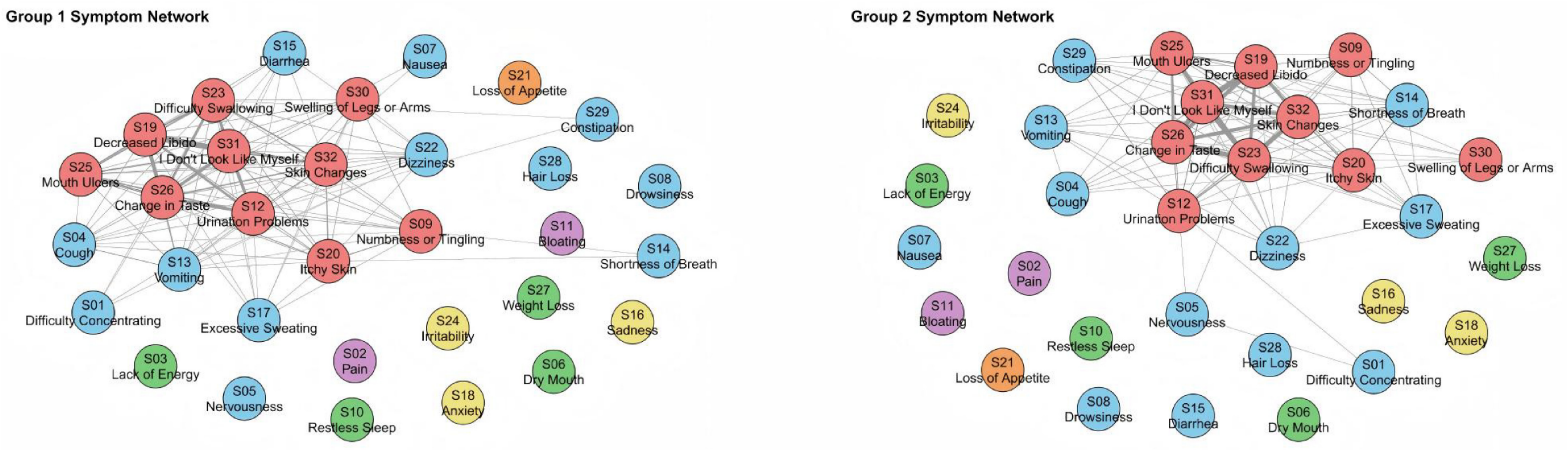
Network diagrams of two subsamples (1 and 2) of 155 subjects. We used automatic random splitting (1:1), with each subset having 77 and 78 subjects, respectively, where we conducted separate DTW analyses. To facilitate visual comparison between the two networks, node placement was aligned using the Procrustes algorithm. The congruence coefficient through 200 random splits was high, with a median of 0.961 (25th and 95th percentiles: 0.947–0.972).

To assess the robustness of the six-cluster solution, the cohort was randomly divided into two subgroups (*n* = 77 and *n* = 78). The clustering structure remained consistent across both samples, confirming the stability and reproducibility of the identified symptom dimensions (Figure 5). Both networks preserved the overall configuration, with the localized physiological perception abnormality cluster consistently displaying strong internal connectivity, underscoring its coherence as a distinct symptom domain. Bootstrapping the random split procedure 200 times yielded a median congruence coefficient of 0.961 (25th and 95th percentiles: 0.947–0.972), indicating high reliability and stability of the symptom structure across individuals.

## 4 Discussion

This study is the first to employ DTW to explore the temporal dynamics of symptom clusters in patients with AP. By integrating idiographic and nomothetic methodologies, we identified substantial inter-individual heterogeneity alongside six relatively stable symptom clusters at the group level. These findings underscore the need to move beyond static, unidimensional scoring systems toward a more dynamic, system-level framework for symptom monitoring in AP.

The emotional disorder cluster comprises symptoms such as S24 (irritability), S16 (sadness) and S18 (anxiety), which reflect emotional instability arising from the psychological burden of illness. These symptoms are frequently precipitated by physical discomfort, fear of disease progression, and uncertainty regarding treatment outcomes. Not only do these emotional symptoms compromise psychological well-being, but they may also adversely affect recovery trajectories. These observations are consistent with prior qualitative studies. For example, Ma et al. (12) noted that patients often experience psychological distress when confronted with unpredictable disease progression, symptom ambiguity, or fear of recurrence. Additionally, patients express a strong desire for emotional support from family, healthcare providers, and broader social networks. Similarly, Chen et al. (44) found that restrictive dietary requirements commonly impede patients’ social engagement, particularly their participation in shared meals, thereby contributing to social isolation and depressive symptoms.

The appetite disorder cluster is typified by appetite-related disturbances, primarily represented by S21 (loss of appetite). Malnutrition is a well-documented complication in AP, often arising from abdominal pain, anorexia, and gastrointestinal dysfunction, and may significantly hinder recovery or even result in mortality. A Swedish qualitative study (45) attributed appetite loss mainly to nausea and vomiting, with several patients continuing to experience reduced appetite and eating difficulties after hospital discharge, contributing to unintentional weight loss. Importantly, appetite loss was perceived not solely as a physical issue but also as a disruptor of social and emotional well-being. Dietary management in AP should therefore be tailored based on disease stage, etiology, and individual needs. Tailored nutritional support is therefore essential: early oral or enteral feeding is recommended where possible, as it is associated with shorter hospital stays and fewer complications (46, 47), with enteral nutrition generally preferred over parenteral routes for better outcomes.

The multi-system physical discomfort cluster comprises a constellation of symptoms affecting various physiological systems, including the digestive, neurological, autonomic, and respiratory systems. The systemic inflammatory response in AP may lead to multi-organ involvement, with organs such as the lungs, kidneys, and liver becoming affected as inflammation progresses (48). Stimulation of nerve endings and intestinal reflexes in early-stage disease often results in nausea and vomiting, while pancreatic exocrine dysfunction can lead to malabsorption and diarrhea. Gastrointestinal motility disorders, frequently associated with fasting, analgesic use, and disease-related pain, contribute to constipation. Anxiety, stemming from the disease itself or the treatment process, exacerbates somatic symptoms and creates a feedback loop that intensifies both physical and psychological distress (44). In severe AP, acute lung injury may manifest early, characterized by dyspnea, cough, wheezing, and respiratory failure. Less commonly reported symptoms, such as alopecia areata, may result from chronic physiological stress, nutritional deficiencies, or metabolic disruption. Given the broad systemic involvement, a holistic understanding of this cluster is imperative for early detection, comprehensive management, and improved patient outcomes.

The localized physiological perception abnormality cluster highlights abnormalities in specific body regions resulting from inflammatory responses, metabolic imbalances, and organ cross-talk, with symptoms such as S25 (mouth ulcers), S23 (difficulty swallowing), S12 (urination problems), S20 (itchy skin), S30 (swelling of legs or arms), S09 (numbness or tingling), S32 (skin changes), S26 (change in taste), S31 (“I don’t look like myself”), S19 (decreased libido). These symptoms often manifest as altered sensory experiences and deteriorations in self-perception. A longitudinal study by Machicado et al. (9) demonstrated that hospitalized AP patients suffer significantly lower health-related quality of life (HRQOL) than the general population, with such detriments persisting even after adjusting for confounding variables. Although many of these symptoms are not life-threatening, they negatively influence nutritional intake, emotional well-being, and patients’ confidence in recovery. Consequently, they merit targeted clinical attention and early intervention.

The functional decline cluster denotes a general decline in physical vitality and homeostatic regulation, involving symptoms such as S10 (restless sleep), S03 (lack of energy), S27 (weight loss), and S06 (dry mouth). Inflammatory processes in AP are known to impair neuropsychiatric regulation, which in turn disrupts sleep patterns and exacerbates fatigue. A decline in energy levels reduces patients’ engagement in daily activities, contributing to emotional disengagement and reduced quality of life. Symptoms such as nausea and vomiting often precede a loss of appetite, culminating in significant weight loss (45). Decreased libido is frequently underreported, potentially due to social stigmatization or patient reticence, and likely exhibits multifactorial etiology driven by systemic inflammation, physical suffering, psychological stress, and emotional detachment. Given its impact on psychosocial functioning, it deserves further clinical and research attention.

Centered on symptoms of S11 (bloating) and S02 (pain), the abdominal discomfort cluster captures the hallmark complaints of AP. Pain, the most common presenting symptom, often prompts emergency care and significantly burdens both physical and psychological domains. Accurate pain assessment using validated instruments is essential for timely and adequate management (49). As a subjective phenomenon, pain is modulated by biological, psychological, and social factors (50), necessitating individualized treatment strategies. In clinical practice, NSAIDs and opioids remain the primary pharmacological options, chosen based on pain intensity and disease severity (51). Abdominal bloating, while often overshadowed by pain, can be particularly distressing and is typically associated with bowel edema, ascites, hematoma, ileus, or visceral distension (52).

Our findings reveal significant interconnections among symptom clusters. For example, emotional disturbances can exacerbate abdominal discomfort. Persistent anxiety may activate the parasympathetic nervous system, resulting in abdominal pain and diarrhea. There is growing evidence that psychological stress alters gut-brain and microbiota-gut-brain axis interactions, thereby amplifying gastrointestinal symptoms (53). These interactions highlight the necessity of integrating psychological support into AP management to address both the physical and emotional dimensions of illness. Our application of DTW to model symptom dynamics in AP aligns with a growing trend in other medical fields. For instance, in depression research, DTW-based analyses have demonstrated that certain symptoms may function as central drivers within symptom networks, thereby influencing the overall clinical trajectory (36). Although the specific symptom profiles differ across diseases, the underlying inter-symptom structure may exhibit common organizational patterns, highlighting the potential of this approach to advance personalized and proactive management strategies across both chronic and acute conditions.

During hospitalization, priority should be given to managing the Abdominal Discomfort cluster, as it directly reflects acute inflammatory activity. Early assessment of the Emotional and Appetite Disorder clusters is also crucial. Given their high out-strength centrality, proactively addressing anxiety and initiating tailored nutrition may prevent downstream symptom exacerbation and reduce persistent symptom risk.

During post-discharge follow-up, the focus shifts toward clusters that drive long-term morbidity, particularly the Multi-system Physical Discomfort and Localized Physiological Perception Abnormality clusters. The Emotional Disorder cluster also remains a critical therapeutic target. Integrating routine psychological screening and support into follow-up care may help disrupt the distress–symptom feedback loop. Similarly, sustained dietary counseling and nutritional monitoring are essential to support recovery and prevent readmission, given the central influence of appetite-related symptoms.

### 4.1 Strengths and limitations

A key innovation of this study lies in the application of DTW to analyze symptom dynamics in AP. Previous investigations have typically focused on static or isolated symptom profiles, often neglecting the temporal symptom interplay. By incorporating both idiographic and nomothetic analyses, we visualized individualized symptom networks and identified symptom interdependencies. These insights enable a more nuanced understanding of symptom trajectories.

However, this study has several limitations. Firstly, a relatively small sample size (*N* = 155) and recruitment from only one tertiary hospital limit the generalizability of this study. Given the known variations in symptom patterns across regions, ethnicities, and age groups, the limited sample size and lack of comparison may introduce a risk of selection bias. Secondly, the undirected DTW approach may inadequately capture relationships between symptoms from different physiological systems. Symptom distances within the same cluster were generally shorter than between clusters. Thirdly, the study’s 12-months follow-up period limited its ability to assess long-term outcomes. Some symptoms potentially persist beyond clinical remission and could continue to affect quality of life. Finally, the study’s single-center recruitment design limits the generalizability of our findings. Participants were recruited from a single tertiary hospital in Changchun, China. And the sample may not be representative of the broader AP population, as it is influenced by the specific demographic, socioeconomic, and clinical characteristics of that particular region and healthcare institution. Although baseline factors were described, statistical adjustments were not performed, and the influence of etiology and comorbidities on symptom trajectories remains to be explored in subsequent analyses.

In summary, by conceptualizing AP as a complex dynamic system, our study employed DTW to map longitudinal symptom trajectories and construct visualized symptom networks. We identified six distinct symptom clusters at the group level and observed notable heterogeneity across individuals. These preliminary findings underscore the potential of personalized symptom management. Future studies with finer temporal resolution are needed to validate and expand upon these insights.

## Data Availability

The original contributions presented in this study are included in this article/Supplementary material, further inquiries can be directed to the corresponding authors.

## Footnotes

1
https://www.R-project.org/
