# Supplementary material for: Time dynamics of symptom progression in patients with acute pancreatitis: a Dynamic Time Warping analysis
Source: Front Med (Lausanne). 2025 Nov 17;12:1703268. doi: 10.3389/fmed.2025.1703268 (PMC12665666; doi:10.3389/fmed.2025.1703268)
Supplement: Supplementary file 1 [file Data_Sheet_1.docx]

Appendix 1

The flowchart of inclusion and exclusion criteria


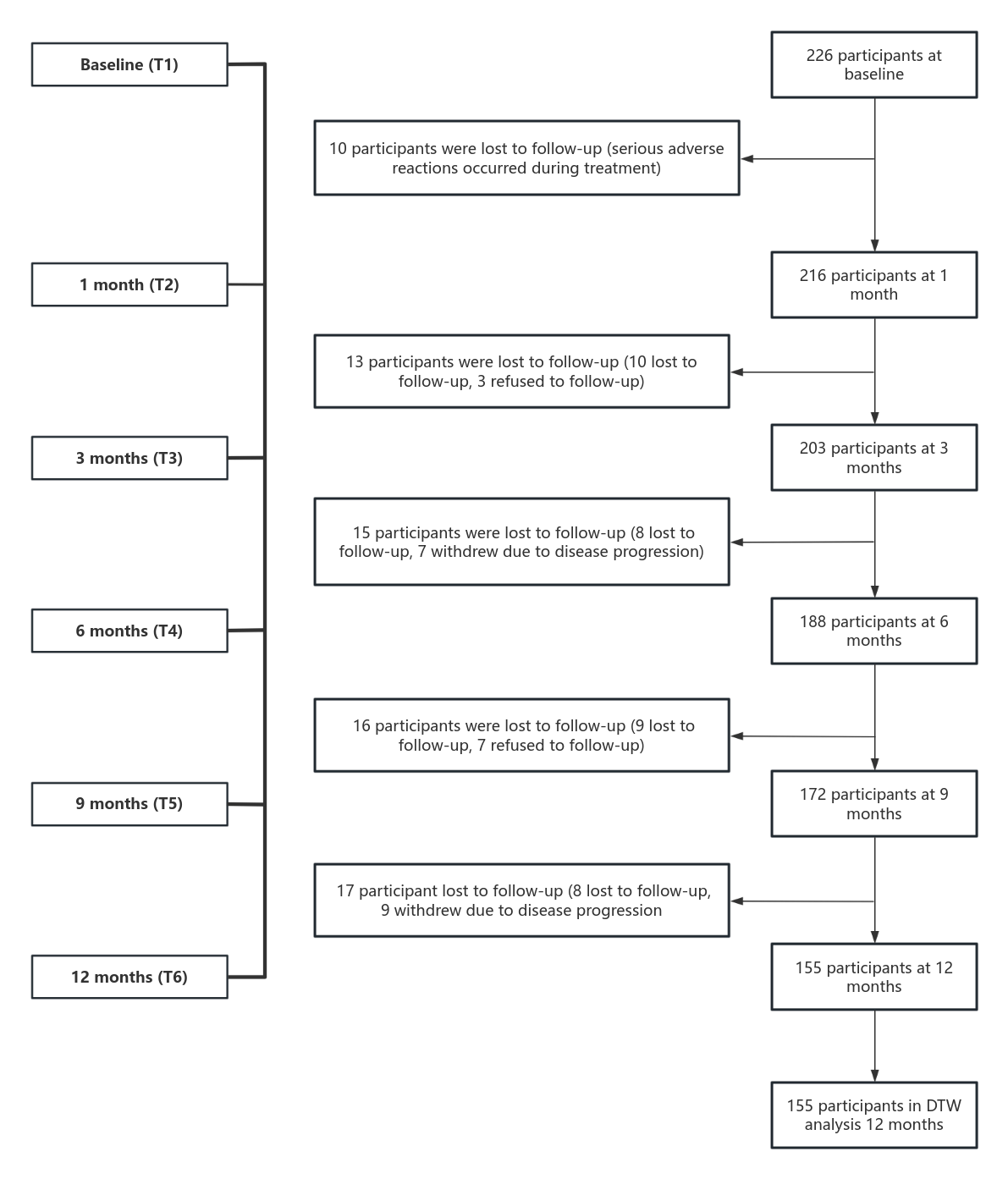


Appendix 2

Baseline information of 3 participants

|  | **A** | **B** | **C** |
| --- | --- | --- | --- |
| **Gender** | Male | Male | Female |
| **Age** | 45 | 36 | 57 |
| **Education Level** | Middle School | High School | High School |
| **Marital Status** | Divorced | Married | Married |
| **Residence Type** | Rural | Urban | Urban |
| **Monthly Income** | ＜1000yuan | ＞5000yuan | ＜1000yuan |
| **Etiology** | Hyperlipidemia | Hyperlipidemia | Encoscopic Retrograde Cholangio -Pancreatography |
| **Severity** | Severe | Moderate | Mild |
| **Smoking History** | Yes | Yes | No |
| **Alcohol Consumption History** | Yes | Yes | No |
| **Diabetes** | Yes | No | No |
| **Cholelithiasis** | Yes | No | Yes |
| **Hyperlipidemia** | Yes | Yes | No |
| **Hospitalization Cost** | ＞10,000yuan | ＜5,0000yuan | ＜5,0000yuan |
| **BMI, kg/m²** | 23.31 | 30.42 | 18.73 |
